# Impact of maternal HIV infection on pregnancy and labor complication and perinatal health outcomes: a South African retrospective study

**DOI:** 10.1007/s00404-026-08347-w

**Published:** 2026-02-19

**Authors:** Zinhle Mlambo, Sapna Ramdin, Randolph Green-Thompson, Jagidesa Moodley, Nalini Govender

**Affiliations:** 1 Department of Basic Medical Sciences, Faculty of Health Sciences, Durban University of Technology, Durban, South Africa; 2 Department of Obstetrics and Gynecology, University of KwaZulu-Natal, Durban, South Africa; 3 Women’s Health and HIV Research Group, Department of Obstetrics and Gynaecology, School of Clinical Medicine, College of Health Sciences, University of KwaZulu-Natal, Durban, South Africa

**Keywords:** COVID-19, Human immunodeficiency virus (HIV), Perinatal birth outcomes, Pregnancy complication

## Abstract

**Background:**

Maternal HIV infection is associated with increased risks of pregnancy complications and adverse perinatal outcomes, particularly in high-prevalence settings like South Africa. The COVID-19 pandemic disrupted healthcare access, potentially exacerbating challenges in antenatal care and HIV management. To our knowledge, limited South African data exist regarding the impact of maternal HIV on birth complications and perinatal birth outcomes especially during the COVID-19 pandemic.

**Aim:**

This study thus evaluates the impact of maternal HIV on pregnancy and perinatal outcomes before and during the COVID-19 pandemic using archived chart records from a tertiary hospital in KwaZulu-Natal, South Africa.

**Methods:**

A retrospective analysis of 8456 birth records from March 2019 to December 2020 was conducted, categorized into pre-pandemic and pandemic periods. Data were stratified by maternal HIV status and analyzed for demographics, antenatal care attendance, ART regimens, labor characteristics, and birth outcomes. Statistical tests, including Chi-square and logistic regression, were used to assess associations between HIV status and outcomes.

**Results:**

Hospital attendance declined during the COVID-19 period, especially among women living with HIV, whose age ranged between 19 and 35 years, and were multigravida, and multiparous. Antenatal care attendance was suboptimal and worsened during the COVID-19 period. ART coverage remained high with maintained viral suppression. Women living with HIV had shorter “active labor” and higher elective cesarean rates during the COVID-19 period. Preterm birth risk was also higher pre-pandemic among women living with HIV but not significantly different during COVID-19 period. Birth weights were lower in HIV-exposed infants pre-pandemic with a non-significant shift during COVID-19 period. Sepsis incidence increased among women living with HIV during COVID-19 period. No maternal deaths were reported.

**Conclusion:**

A decline in hospital attendance was noted during the COVID-19 period among women living with HIV, with antenatal care attendance being suboptimal and exacerbated. Maternal HIV remains a critical factor influencing birth outcomes, necessitating sustained focus on tailored care during crises to protect vulnerable populations.

## Introduction

Globally, 40.8 million people were reported to be living with human immunodeficiency virus (HIV) in 2024, of which [1] 53% were females, and of reproductive age [1]. HIV infection is a major contributor to maternal mortality and morbidity, especially in low- and middle-income countries (LMIC), particularly sub-Saharan Africa [2] and southern Asia [3]. Pregnant women living with HIV have two-to-ten times increased risk of death during pregnancy and the postpartum period compared with uninfected pregnant women [4, 5]. South Africa (SA) remains the epicenter of HIV infection, with approximately 8 million people living with HIV (PLHIV) [1]. Of note, in 2020, it was reported that one in three pregnant women receiving antenatal care in SA is HIV infected [6].This infectivity rate accounts for 27.5% of most early maternal deaths in SA, second to hypertensive disorders of pregnancy (HDPs) [6, 7].

Maternal HIV infection is also associated with increased pregnancy complications, such as preterm birth, low birthweight, IUGR, anemia, and hypertensive disorders, and higher risk of adverse perinatal outcomes including low birth weight (LBW: < 2500 g) and perinatal and early neonatal mortality [8–10]. The effect of antiretroviral therapy (ART) on this association remains unclear [11]. It is well established that ART improves the overall health and quality of life for pregnant women living with HIV and reduces the rate of mother-to-child transmission [12–14]. However, its use during pregnancy may be associated with preterm birth, stillbirth, and fetal growth restriction, depending on ART regimen and the timing of initiation [15–17].

Despite the immediate rollout of the World Health Organisation (WHO) Option B+ strategic initiation of lifelong ART to all South African pregnant and breastfeeding women living with HIV in 2015, regardless of HIV staging or CD4^+^count [18, 19]. HIV remains the primary cause of morbidity and mortality for children under 5 years [20, 21]. In SA, the maternal and perinatal mortality and morbidity burden was further compounded by the COVID–19 pandemic [22, 23]. Additionally, the nationwide lockdown implemented between March 2020 and July 2022 due to rapid community transmission of SARS CoV-2, resulted in disruptions in daily transportation reduced community services and healthcare access [24, 25]. This subsequently created difficulties in managing chronic diseases, such as HIV, diabetes, and hypertension; negatively impacting maternal and perinatal health, particularly those affected with HIV[26].

To our knowledge, limited South African data exist regarding the impact of maternal HIV on birth complications and perinatal birth outcomes, especially during the COVID-19 pandemic. In view of this, we evaluated the effect of maternal HIV infection on pregnancy complications and perinatal health outcomes using archived data from a local tertiary hospital in KwaZulu-Natal.

## Materials and methods

### Ethical approval

This study was approved by the Institutional Research Ethics Committee, Durban University of Technology (IREC 054/22). There was no direct patient involvement during data collection as only archived data records were used.

### Study details

This retrospective study was a primary analysis of birth registry data extracted from a local tertiary affiliated hospital in KwaZulu-Natal, South Africa. Only data between 1 March 2019 and 31 December 2020 were included irrespective of maternal health status or perinatal birth outcomes. All recorded data were manually extracted and thereafter coded for statistical analysis. Most outcomes and covariates were directly extracted from structured data fields within the birth registry record. Data extraction was conducted by trained research assistants supervised by the PI. Data were compared based on time periods categorized as pre**-**COVID-19 (March 2019–February 2020) and during the COVID-19 (March 2020–December 2020) pandemic. All data were sub-categorized according to HIV status (HIV-negative and HIV-positive); age (< 18, 19–35 and > 35 years old); gravidity (primigravida or multigravida); parity (nulliparous, primiparous, and multiparous); and gestational age (at birth as < 37 weeks and ≥ 37 weeks), respectively. Antenatal care (ANC) bookings were classified as < 20 weeks and > 20 weeks; and number of ANC visits as < 8 and ≥ 8, respectively. ART treatment was categorized as either untreated, fixed-dose combination (FDC) or triple ART treatment, whereas viral load as LTDL (undetectable), ≤ 1000 (suppressed), > 1000 (unsuppressed), and > 10,000 copies, respectively.

Mode of delivery was categorized as vaginal birth (normal vertex) and cesarean section (CS), with CS further sub-categorized as emergency CS and elective CS, respectively. Regarding labor, the following categories were analyzed, viz., follows, vacuum utilization, forceps utilization, breech presentation, miscarriage, induction of labor, antepartum hemorrhage, postpartum hemorrhage, preeclampsia (PE), eclampsia, sepsis, and obstructed and/or prolonged labor. Perinatal health outcomes were categorized as live and stillbirths, with stillbirths sub-categorized as macerated stillbirth (MSB) and fresh stillbirth (FSB). The APGAR (5 min) and (10 min) scores were categorized as low (0–3), intermediate (4–6), and normal (7–10). The variables “resuscitate bag and mask utilization”, “neonatal birth defects”, “birth defects defined”, Nevirapine (NVP), and daily NVP administration were categorized as “yes”, “no”, and “non-applicable (N/A)”, respectively.

### Statistical analysis

All data were analyzed using STATA (version 12, STATACORP) and GraphPad Prism 5. The data were assessed for normality using the skewness and kurtosis normality test. All continuous data are summarized as means and standard deviation. The unpaired t test was used to compare the means for maternal age, gravidity, parity, and gestational age between the pre-COVID-19 and COVID-19 pandemic time periods. Categorical data are presented as frequency and percentages, and the Pearson’s chi-squared test was used to determine bivariate associations between demographic and clinical variables stratified by time (i.e., pre-COVID-19 and during COVID-19 period). Binary logistic regression was used to determine the relationship between HIV status and gestational age at birth, cesarean section delivery, stillbirth, birth weight, and 5- and 10-min APGAR scores during both time periods. A 95% confidence interval (CI) was used and *p* < 0.05 was considered statistically significant.

## Results

### Maternal characteristics

Table 1 illustrates the demographic and clinical characteristics during both study periods regardless of HIV status. Parity differed significantly between the pre-pandemic and COVID-19 periods; however, multigravida was most prominent in both periods. Access to antenatal care remained high. Although timely ANC uptake and the percentage of women completing ≥ 8 visits dropped slightly during the pandemic. During COVID-19, the mean gestational age at birth declined, and preterm births was significantly reduced compared to the pre-pandemic period. There was a significant reduction in both normal vaginal deliveries and emergency cesarean sections during the COVID-19 pandemic versus the pre-pandemic period. As expected, elective cesarean deliveries increased. Live births decreased slightly during the pandemic, and insignificant differences were noticed in stillbirths and birth defects. Nevertheless, the rates of maternal complications, viz., postpartum hemorrhage, preeclampsia, sepsis, and prolonged labor, were significantly greater during the pandemic, in contrast to eclampsia.

**Table 1:** Demographic and clinical characteristics of the study population

Demographic/clinical features	Pre–COVID–19 (*n* = 4866)	COVID–19 pandemic (*n* = 3607)	*p* value
Maternal characteristics			
Age (mean ± SD)	26.97 ± 6.73	26.90 ± 7.62	0.080
Age category (years)			
< 18	508 (10.48%)	436 (12.12%)	0.020*
19–35	3752 (77.39%)	2695 (74.94%)	
> 35	588 (12.13%)	465 (12.93%)	
Gravidity			
Primigravida	1462 (30.13%)	1134 (31.75%)	0.110
Multigravida	3391 (69.87%)	2438 (68.25%)	
Parity			
Nulliparous	1444 (29.81%)	1150 (32.28%)	0.010*
Primiparous	1468 (30.31%)	1107 (31.07%)	
Multiparous	1932 (39.88%)	1306 (36.65%)	
ANC booking			
< 20 weeks	2455 (51.48%)	1747 (49.62%)	0.090
> 20 weeks	2314 (48.52%)	1774 (50.38%)	
Number of ANC visits	5.16 ± 2.32	5.11 ± 2.29	0.350
< 8	4193 (87.90%)	3099 (89.08%)	0.100
≥ 8	577 (12.10%)	380 (10.92%)	
Gestational age at birth	37.45 ± 3.74	37.02 ± 4.10	0.001*
< 37 weeks	1044 (21.49%)	899 (24.97%)	0.001*
≥ 37 weeks	3815 (78.51%)	2702 (75.03%)	
Birth details			
Mode of birth			
Normal vertex	2490 (51.17%)	1742 (48.29%)	0.001*
Emergency CS	2068 (42.50%)	1493 (41.39%)	
Elective CS	308 (6.33%)	372 (10.31%)	
Live births	4705 (96.69)	3493 (96.84%)	0.001*
Still births (total)	160 (3.29%)	114 (3.16%)	
Macerated stillbirth	132 (2.71%)	98 (2.72%)	0.630
Fresh stillbirth	28 (0.58%)	16 (0.44%)	
Birth defects			
Yes	33 (0.68%)	20 (0.56%)	0.005*
N/A	58 (1.19%)	74 (2.06%)	
Obstructed/prolonged childbirth	35 (0.72%)	71 (1.98%)	0.001*
Maternal complications			
Postpartum hemorrhage	27 (0.56%)	70 (1.95%)	0.001*
PE	62 (1.28%)	97 (2.70%)	0.001*
Eclampsia	30 (0.62%)	16 (0.45%)	0.290
Sepsis	56 (1.15%)	348 (9.37%)	0.001*

*ANC* antenatal care, *CS* cesarean section, *N/A* not applicable

^*^p < 0.05; **p < 0.005; ***p < 0.001 are considered statistically significant

Additionally, the demographic and clinical profiles stratified by HIV status and study period (pre-COVID-19 and during COVID-19) are shown in Table 2. Of the 8456 records analyzed, 4826 pregnant women attended the hospital prior to the pandemic, versus 3630 during COVID-19 period and/or national lockdown. Of those who attended during COVID-19 period (pre-COVID), 1842 were HIV-infected; this number declined (*n* = 1142) during COVID-19 period. Noteworthy, significant statistical differences were observed for maternal age, gravidit**y,** and parity categories (*p* < 0.001) between the two periods analyzed. Among the HIV-infected women living with HIV who accessed the hospital pre- and during COVID-19, the majority were aged between 19 and 35 years (77.09% *vs* 74.96%), multigravida (85.97% *vs* 84.87%) and multiparous (55.59% *vs* 50.97%). A similar trend was observed among the HIV-uninfected women for age and gravidity; however, for parity, nulliparous (39.38% *vs* 43.45%) was dominant in both time periods. A noticeable decline was noted in the number of ANC bookings made prior to 20 weeks of gestation by HIV-positive women versus HIV-uninfected women during both time periods (pre-COVID-19 1415 *vs* 885; pandemic 1083 *vs* 690), albeit non-significant. Notably, the average number of ANC visits were less regardless of HIV status or the time periods analyzed. Only 330 (11.21%) HIV-uninfected women attended more than eight ANC visits before the pandemic. For HIV-infected women, 245 (13.56%) accessed more than eight ANC visits and this access decreased during COVID-19 period.

**Table 2:** Demographic and clinical profiles stratified by HIV status and study period (pre-COVID-19 and during COVID-19)

	Pre-COVID–19 (n = 4826)			COVID–19 (n = 3630)		
	HIV status		*p* value	HIV status		*p* value
	Negative (*n* = 2984)	Positive (*n* = 1842)		Negative (*n* = 2188)	Positive (*n* = 1142)	
Age						
< 18	454 (15.21%)	50 (2.71%)	< 0.001*	348 (15.90%)	88 (6.2%)	< 0.001*
19–35	2315 (77.58%)	1420 (77.09%)		1638 (74.86%)	1052 (74.96%)	
> 35	215 (7.21%)	372 (20.20%)		202 (9.23%)	2.63 (18.75%)	
Gravidity						
Primigravida	1203 (40.06%)	259 (14.03%)	< 0.001*	922 (42.43%)	211 (15.13%)	< 0.001*
Multigravida	1800 (59.94%)	1587 (85.97%)		1251 (57.57%)	1184 (84.87%)	
Parity						
Nulliparous	1174 (39.38%)	264 (14.33%)	< 0.001*	942 (43.45%)	207 (14.88%)	< 0.001*
Primiparous	904 (30.33%)	554 (30.08%)		630 (29.06%)	475 (34.15%)	
Multiparous	903 (30.29%)	1024 (55.59%)		596 (27.49%)	709 (50.97%)	
ANC booking						
< 20 weeks	1415 (48.24%)	885 (48.76%)	0.310	1083 (50.61%)	690 (50.07%)	0.570
> 20 weeks	1518 (51.76%)	930 (51.24%)		1057 (49.39%)	688 (49.93%)	
No of ANC visits						
< 8	2613 (88.79%)	1562 (86.44%)	0.050*	1904 (88.97%)	1191 (89.21%)	0.820
≥ 8	330 (11.21%)	245 (13.56%)		380 (10.92%)	144 (10.79%)	
Gestational age at birth						
< 37 weeks	601 (20.07%	437 (23.70%)	0.040*	537 (24.53%)	360 (25.59%)	0.660
≥ 37 weeks	2392 (79.93%)	1407 (76.30%)		1652 (75.47%)	1047 (74.41%)	

*p < 0.05; **p < 0.005; ***p < 0.001 considered statistically significant

A significant difference was noted in the number of HIV-positive women who were on ART during both time periods (*p* < 0.001). Of note, most women were on either dual (FDC) (5.55%) or triple (91.40%) ART prior to the pandemic, whereas during COVID-19 period, only 133 (9.45%) were managed on FDC and 1209 (85.93%) on triple ART. Regrettably, a small percentage of HIV-positive women were not managed on any form of ART prior (3.05%) to or during (4.62%) the pandemic. Noteworthy, viral loads of > 1000 copies in this cohort were lower during COVID-19 period compared to prior. Likewise, 5.74% of this cohort demonstrated > 10,000 copies during COVID-19 period versus 4.75%. Most deliveries occurred at ≥ 37 weeks regardless of the time periods analyzed. However prior to the pandemic, preterm (< 37 weeks) deliveries were higher recorded among HIV-uninfected (*n* = 601) versus infected women (*n* = 437; *p* < 0.05). A similar trend was noted during COVID-19 period (537 uninfected *vs* 360 HIV infected).

### Labor and birth outcomes

The labor and birth outcomes for the study population are shown in Table 3. The duration of active labor was significant shorter among the HIV infected versus the uninfected women prior *p* < 0.001 and during *p* < 0.005 the pandemic. During COVID-19 period, lower rates of normal vertex and emergency CS deliveries were observed in HIV-infected women versus pre-COVID-19. As expected, elective CS deliveries increased during COVID-19 period in both women living with HIV and uninfected women compared to pre-COVID. Notably, low rates were noted for vacuum utilization during both time periods, with a slight decrease in HIV-infected vs HIV-uninfected women during COVID-19 period. The use of forceps in supporting the delivery was significant higher in HIV-uninfected versus HIV-infected women during COVID-19 period (1.15% vs 0.07%; *p* = 0.001). During COVID-19 period, a reduction was observed in breach presentation, induction of labor, and miscarriage in the HIV-infected women compared to prior. Fortunately, no maternal deaths were recorded during both time periods. The number of live births was decreased in both HIV-infected and uninfected women during both time periods analyzed. Prior to the pandemic, similar reductions were noted for stillbirths in both HIV-infected (*n* = 57 *vs* 47) and uninfected (*n* = 72 *vs* 59) women compared to during COVID-19 period.

**Table 3:** Labor and birth outcomes stratified by HIV status and study period (pre-COVID-19 and during COVID-19)

Labor and birth outcomes	Pre-COVID–19 (*n* = 4826)			COVID–19 (*n* = 3630)		
	HIV status		*p* value	HIV status		*p* value
	Negative (*n* = 2984)	Positive (*n* = 1842)		Negative (*n* = 2188)	Positive (*n* = 1142)	
Duration of active labor (mean, SD)	9.12 (4.51)	8.11 (4.64)	< 0.001*	9.28 (4.63)	8.57 (4.28)	0.004*
Mode of birth						
Normal vertex	1536 (51.01%)	951 (51.43%)	0.820	1063 (48.41%)	675 (47.97%)	0.160
Emergency CS	1281 (42.54%)	784 (42.40%)		923 (42.03%)	570 (40.51%)	
Elective CS	194 (6.44%)	114 (6.17%)		210 (9.56%)	162 (11.51%)	
Assisted vaginal delivery						
Vacuum utilization	1 (0.03%)	0 (0.00%)	< 0.001*	4 (0.18%)	1 (0.07%)	0.680
Forceps utilization	10 (0.34%)	7 (0.39%)	0.940	25 (1.15%)	1 (0.07%)	0.001*
Breech presentation	13 (0.45%)	12 (0.68%)	0.560	2 (0.09%)	1 (0.08%)	0.990
Induction of labor	115 (3.84%)	68 (3.69%)	0.880	45 (2.06%)	24 (1.71%)	0.750
Miscarriage	6 (0.21%)	6 (0.33%)	0.680	2 (0.09%)	3 (0.22%)	0.620
Maternal death	0 (0.00%)	0 (0.00%)		0 (0.00%)	0 (0.00%)	
Live births	2909 (97.10%)	1773 (95.94%)	0.080	2079 (94.72%)	1333 (94.74%)	0.970
Stillbirth (total)	72 (2.39%)	57 (3.08%)	0.130	59 (2.69%)	47 (3.35)	0.100
MSB	63 (2.09%)	49 (2.65%)	0.340	52 (2.37%)	42 (2.99%)	0.520
FSB	9 (0.30%)	8 (0.43%)		7 (0.32%)	5 (0.36%)	
Resuscitation bag and mask	57 (1.91%)	36 (1.95%)	0.960	18 (0.82%)	1 (0.07%)	0.060
APGAR (5 min)						
Low (0–3)	135 (4.50%)	87 (4.72%)	0.920	153 (6.97%)	70 (5.00%)	0.003*
Intermediate (4–6)	124 (4.13%)	79 (4.28%)		96 (4.38%)	40 (2.86%)	
Normal (7–10)	2739 (91.27%)	1677 (90.94%)		1944 (88.65%)	1289 (92.14%)	
APGAR (10 min)						
Low (0–3)	109 (3.64%)	74 (4.02%)	0.600	136 (6.20%)	62 (4.43%)	0.020*
Intermediate (4–6)	45 (1.50%)	19 (1.03%)		26 (1.19%)	9 (0.64%)	
Normal (7–10)	2843 (94.83%)	1749 (94.90%)		2032 (92.62%)	1328 (94.92%)	
Birth weight (mean, SD)	2.97 (0.74)	2.90 (0.75)	0.002*	2.94 (0.76)	2.90 (0.79)	0.160
Extremely low	87 (2.89%)	61 (3.30%)	0.050*	49 (2.24%)	45 (3.21%)	0.410
Very low	107 (3.55%)	68 (3.68%)		119 (5.44%)	77 (5.50%)	
Low	308 (10.23%)	226 (12.23%)		262 (11.97%)	169 (12.07%)	
Normal	2390 (79.40%)	1442 (78.03%)		1680 (76.78%)	1052 (75.14%)	
High	118 (3.92%)	51 (2.76%)		78 (3.56%)	57 (4.07%)	
Birth defects						
Yes	19 (0.63%)	14 (0.76%)	0.700	13 (0.60%)	7 (0.50%)	0.230
N/A	41 (1.37%)	17 (0.92%)		52 (2.38%)	22 (1.57%)	
Maternal complications						
Antepartum hemorrhage	46 (1.54%)	36 (1.95%)	0.500	29 (1.33%)	25 (1.79%)	0.540
Postpartum hemorrhage	17 (0.57%)	10 (0.54%)	0.960	44 (2.01%)	26 (1.85%)	0.940
Preeclampsia	44 (1.47%)	18 (0.97)	0.300	67 (3.06%)	30 (2.14%)	0.250
Eclampsia	19 (0.64%)	11 (0.60%)	0.950	15 (0.68%)	1 (0.07%)	0.030*
Sepsis	25 (0.84%)	31 (1.68%)	0.030*	174 (7.96%)	174 (12.52%)	< 0.001*
Obstructed/Prolonged labor	26 (0.87%)	9 (0.49%)	0.300	52 (2.38%)	19 (1.37%)	0.260

*p < 0.05; **p < 0.005; ***p < 0.001 considered statistically significant

Prior to the pandemic, resuscitation was only required for a small number of HIV-uninfected versus infected babies and decreased during COVID-19 period in both groups, the 5- and 10-min APGAR scores ranged between 7 and 10 for most babies born regardless of the maternal HIV status or the period analyzed). During COVID-19 period, a significant decline was noted for normal APGAR scores at 5 min (*n* = 1289 *vs* 1944; *p* < 0.005) and 10 min (*n* = 1328 *vs* 2032; *p* < 0.05), for babies born to HIV-infected mothers versus uninfected mothers, respectively. A higher percentage of babies were born from HIV-uninfected mothers with APGAR scores in the low range (i.e., 5 min: 6.97% *vs* 5.00%; *p* < 0.005; 10 min: 6.20% *vs* 4.43%; *p* < 0.05) and intermediate ranges (5 min: 4.38% *vs* 2.86%; *p* < 0.005; 10 min: 1.19% *vs* 0.64%; *p* < 0.05) compared to HIV-infected women.

Prior to the pandemic, birth weight was significant lower (2.90 ± 0.75 *vs* 2.97 ± 0.74; *p* < 0.005) in babies born to HIV-infected compared to uninfected mothers; and the number of live births was also significant lower 1422 (77.16%) compared to during COVID-19 period 555 (39.61%); *p* < 0.001. Of these births, a significant decline was noted among babies receiving Nevirapine postpartum 1323 (71.86%) *vs* 546 (38.97%); *p* < 0.001 and those receiving daily doses 359 (19.53%) *vs* 262 (18.70%); *p* < 0.001, prior to and. Noteworthy, the rate of eclampsia development was lower in HIV-infected pregnancies during COVID-19 period uninfected pregnancies (0.07% *vs* 0.68%; *p* < 0.05). During COVID-19 period, sepsis infection was higher in HIV-infected pregnancies compared to uninfected pregnancies (12.52% *vs* 7.96%; *p* < 0.001).

### Association between HIV and infant birth outcomes during the COVID-19 pandemic

Significant associations were observed between HIV status and birth outcomes during the COVID-19 pandemic (Table 4). Prior to the pandemic, HIV-positive women had (OR: 1.23; 95% CI: 1.07, 1.41; *p* = 0.004) significantly higher risk of delivery at ≤ 37 weeks. In contrast, this risk was reduced to 1 (OR: 1.06; 95% CI: 0.91, 1.23; *p* = 0.47) likelihood during COVID-19 period, albeit non-significant. Additionally, HIV-positive women were less likely to deliver at ≥ 37 weeks during the pre-COVID–19 (OR: 0.81; 95% CI: 0.71, 0.94 *vs* OR: 1.23; 95%CI: 1.07, 1.41; *p* = 0.004) and COVID-19 (OR: 0.94; 95% CI: 0.81, 1.10 *vs* OR: 1.06; 95% CI: 0.91, 1.24; *p* = 0.47) periods in comparison to HIV-uninfected women. Despite non-significance, HIV-positive women had lower risk of requiring emergency cesarean section (C/S) during the pandemic. However, the likelihood of elective C/S during this period among HIV-positive women was high. Moreover, a higher odds of stillbirth was associated with positive HIV status, with a (OR: 1.28; 95% CI: 0.93, 1.76; *p* = 0.13) and (OR: 1.37; 95% CI: 0.94, 1.99; *p* = 0.10) times likelihood of stillbirth during the pre-COVID-19 periods, respectively. In contrast, HIV-uninfected women had a lower odds of stillbirth prior to (OR: 0.78; 95% CI: 0.57, 1.08; *p* = 0.13) and during (OR: 0.73; 95% CI: 0.50, 1.06; *p* = 0.10) the pandemic.

**Table 4:** Associations between HIV status and labor and birth outcomes

	HIV negative				HIV positive			
	Pre-COVID-19 (*n* = 3011)		COVID (*n* = 2196)		Pre-COVID-19 (*n* = 1849)		COVID (*n* = 1407)	
	OR (95% CI)	*p* value	OR (95% CI)	*p* value	OR (95% CI)	*p* value	OR (95% CI)	*p* value
Gestational age at birth:								
< 37 weeks	1.06 (0.91, 1.24)	0.470	0.94 (0.81, 1.10)	0.470	1.23 (1.07, 1.41)	0.004**	1.06 (0.91, 1.23)	0.470
> 37 weeks	1.23 (1.07, 1.41)	0.004**	1.06 (0.91, 1.24)	0.470	0.81 (0.71, 0.94)	0.004**	0.94 (0.81, 1.10)	0.470
Cesarean section								
Emergency	1.01 (0.90, 1.14)	0.870	1.02 (0.89, 1.19)	0.700	0.99 (0.88, 1.11)	0.840	0.97 (0.84, 1.12)	0.700
Elective	1.05 (0.82, 1.35)	0.680	0.82 (0.66, 0.13)	0.090	0.95 (0.74, 1.21)	0.680	1.21 (0.97, 1.52)	0.090
Stillbirth	0.78 (0.57, 1.08)	0.130	0.73 (0.50, 1.06)	0.100	1.28 (0.93, 1.76)	0.130	1.37 (0.94, 1.99)	0.100
Birth weight								
Extremely low	–		0.70 (0.43, 1.16)	0.170	–		1.42 (0.86, 2.33)	0.170
Very low	1.10 (0.71, 1.72)	0.670	–		0.91 (0.58, 1.42)	0.670	–	
Low	0.96 (0.66, 1.38)	0.810	1.00 (0.71, 1.41)	0.990	1.04 (0.72, 1.51)	0.810	1.00 (0.71, 1.41)	0.990
Normal	1.16 (0.83, 1.62)	0.380	1.03 (0.77, 1.39)	0.830	0.86 (0.62, 1.20)	0.370	0.97 (0.72, 1.30)	0.830
High	1.62 (1.02, 2.58)	0.040*	0.87 (0.56, 1.37)	0.560	0.62 (0.39, 0.98)	0.040*	1.14 (0.73, 1.79)	0.560
APGAR 5 min								
Low	0.52 (0.05, 5.05)	0.570	0.91 (0.57, 1.45)	0.690	1.93 (0.20, 18.89)	0.570	1.10 (0.69, 1.75)	0.690
Intermediate	0.52 (0.05, 5.12)	0.580	–		1.91 (0.20, 18.70)	0.580	–	
Normal	0.54 (0.06, 5.23)	0.600	0.63 (0.43, 0.92)	0.020*	1.83 (0.19, 17.67)	0.600	1.59 (1.09, 2.31)	0.020*
APGAR 10 min								
Low	0.62 (0.34, 1.15)	0.130	0.76 (0.34, 1.72)	0.510	1.61 (0.87, 2.97)	0.130	1.32 (0.58, 2.98)	0.510
Intermediate	–		–		–		–	
Normal	0.69 (0.40, 1.18)	0.170	0.53 (0.25, 1.13)	0.100	1.46 (0.85, 2.50)	0.170	1.89 (0.88, 4.04)	0.100

*p < 0.05; **p < 0.005; ***p < 0.001 considered statistically significant

Babies born to HIV-positive women were less likely to be within the normal birth weight range during COVID-19 period in comparison to HIV-negative women. During COVID-19 period, the risk of delivering extremely LBW babies is almost doubled in HIV-positive pregnant women compared to HIV-negative women. Interestingly, during COVID-19 period, HIV-positive women were more likely to deliver a baby of high birth weight, compared to HIV-negative women. During COVID-19 period, babies born to HIV-positive mothers were more likely to produce low 5 min APGAR scores in comparison to HIV-uninfected women during the pre-COVID-19 and lockdown. However, it is interesting to note that during COVID-19 period, HIV-positive pregnant women also demonstrated a significant 1.5-fold increase (OR: 1.59; 95% CI: 1.09, 2.31; *p* = 0.02) in the odds of delivering babies that had a normal 5 min APGAR score in comparison to HIV-negative women, where the odds of this was significantly reduced (OR: 0.63; 95% CI: 0.43, 0.92; *p* = 0.02). A similar trend was followed with 10 min APGAR scores, where babies born to HIV-positive women had a higher likelihood of low APGAR scores compared to those of HIV-negative mothers during the pre-COVID-19 and the COVID-19. Of interest, normal 10 min APGAR scores were also more likely to be associated with babies born from HIV-positive in comparison to HIV-negative women during the pre-COVID-19 and COVID-19 periods.

## Discussion

The significant differences observed between the pre-COVID-19 and COVID-19 periods are consistent with emerging global evidence demonstrating the pandemic impact on maternal and perinatal outcomes [27]. Increases in adolescent and nulliparous pregnancies during the COVID-19 may reflect disruptions in access to reproductive and antenatal services, a trend similarly reported in other low- and middle-income settings [28]. The reduction in gestational age and rise in preterm births align with study that showed an elevated risks of preterm delivery associated with pandemic-related stress, reduced ANC attendance, and delayed care-seeking [29]. The higher rates of complications including obstructed labor, postpartum hemorrhage, preeclampsia, and sepsis mirror reports of strained health systems leading to compromised intrapartum care and delayed management of obstetric emergencies [29, 30]. Our findings also show significant differences in maternal demographic and clinical characteristics among pregnant women living with HIV between the pre-COVID-19 and COVID-19 periods. Hospital attendance decreased during COVID-19 period, consistent with global reports of reduced maternal health service use driven by movement restrictions, fear of infection, and shifts in health system priorities [30, 31]. Albeit this reduction was higher among HIV-infected women, confirming the adverse impact of the pandemic on access to HIV care and antenatal services, as previously reported in sub-Saharan Africa [32]. Our findings also highlight a statistically significant difference for maternal age, gravidity, and parity between the two study periods (*p* < 0.001). Most of HIV-infected women were aged 19–35 years and were predominantly multigravida and multiparous, consistent with the previous reports that women of reproductive age with extensive obstetric histories are at an elevated risk for HIV acquisition and transmission [33–35].

Noteworthy, HIV infection is associated with a higher cumulative childbirth and sexual exposure, whereas antenatal care booking patterns remained suboptimal for HIV-infected women, with persistent late bookings delaying the timely initiation of prevention of vertical transmission of HIV interventions, thus corroborating the previous reports [36]. Alarmingly, most women did not meet the WHO recommended minimum of eight ANC visits, a deficit that worsened during COVID-19 period, reflecting ongoing challenges in maternal health service delivery and utilization [37, 38].

Regarding the ART regimen data, a slight increase was observed in dual fixed-dose combination (FDC) ART usage in contrast to a decrease in triple ART regimens among HIV-infected women. This discrepancy may be due to the implementation of a more simplified treatment protocol, or public health modifications, and/or revised guidelines endorsing dual therapy, all of which may have focused on ensuring continuity of ART while minimizing COVID-19 exposure risk [39]. Despite these disruptions, there was a notable reduction in the proportion of pregnant HIV-infected pregnant women with elevated viral loads (> 1000 copies/mL and > 10,000 copies/mL), which may be attributed to effective maintenance of virologic suppression among those retained in care.

Birth outcomes showed that majority of women delivered at term (≥ 37 week gestation) in both study intervals. Our findings corroborate Molina et al. (2022), who reported a stabilization in the rate of preterm births before and during COVID-19 [40]. We also observed a reduction in the number of preterm births during COVID-19 period, which aligns with the previous reports [41, 42]; however, this may be attributed to reduced working hours, decreased physical and/or emotional stress associated with employment, a decrease in infections due to improved hygiene and reduced social interactions, lower rates of smoking and substance abuse, the presence of familial support at home, increased time for physical exercise, reduced exposure to environmental pollutants, and less vehicular travel, resulting in decreased stress and fewer accident [41, 42].

Additionally, we demonstrate significantly shorter duration of active labor in women with HIV compared to women without, both before and after the COVID-19 pandemic. Our findings corroborate the previous reports, which indicate that prolonged labor increases the risk of mother-to-child HIV transmission, HIV-infected women typically have shorter active labor periods than uninfected women [43–45]. Current clinical management plans strive to reduce the duration of labor as a means to reduce fetal exposure to the virus through maternal blood and cervical secretions [43]. More recently, an association between HIV infection and changes in myometrial function and/or hyperinflammatory activity was reported, suggestive of an ameliorative effect on labor progression[46]. Overall, data regarding modes of delivery failed to demonstrate any significance between the two study intervals; however, cesarean section rates increased during COVID-19 period for both HIV-infected and uninfected women. The low rates of vacuum and forceps deliveries are consistent with longstanding limitations in many African settings [47], which was further decreased during COVID-19 period perhaps due to concerns about prolonged close contact with potentially infectious patients and staff shortages.

During COVID-19 period, we observed a slight decrease in breech presentations, labor inductions, and miscarriages among HIV-infected women, and no maternal deaths were reported in either group across both study intervals. These findings suggests that the local healthcare systems strived to maintain high standards of maternal care during and after the pandemic, irrespective of HIV status. Both live births and stillbirths decreased during the COVID-19 pandemic, which is inconsistent with that reported by Galis and co-authors, who demonstrated an increase in the stillbirth rate during COVID-19 pandemic (RR: 1.53, 95% CI, 1.05–2.23) [48]. The data from prior to the pandemic highlight normal APGAR scores between 5 and 10 min after birth as well as a reduction in newborn resuscitation rates regardless of the mother’s HIV status. In contrast, during COVID-19 period, fewer HIV-infected women had babies with normal APGAR scores, while HIV-uninfected women had comparatively lower or intermediate scores.

Birth weight was also significantly lower among infants exposed to HIV before the pandemic, a trend widely reported in the literature and attributed to HIV-associated placental dysfunction, chronic inflammation, and ART exposure [8, 49, 50]. During COVID-19 period, obstetric complications like hemorrhage, preeclampsia, and obstructed labor were less common in women infected with HIV than those not infected. However, our findings differs from the previous reports that highlight higher complication rates in HIV-infected pregnancies [47, 51–53], indicative of HIV-infected women practicing different health-seeking practices, visiting facilities, or changes in risk profiles [54].

During COVID-19 period, we also demonstrated a higher incidence of sepsis among women with HIV. Studies indicate that HIV infection can induce immunosuppression, subsequently resulting in a broader spectrum of etiologies that can promote the onset of sepsis [55, 56]. Prior to the pandemic, we report a higher of preterm delivery (≤ 37 weeks) among HIV-infected women, which concur with Naidoo and co-authors, who reported that HIV-infected were significantly more ~ fourfold likely to have preterm deliveries than their HIV-uninfected counterparts [57]. Additionally, reports confirm a higher risk of Preterm Birth < 37 weeks among HIV-infected women than those without (32% vs. 23%, RR = 1.43, 95% Cl: 1.07–1.91) [58]. However, our bivariate analysis showed no correlation between maternal HIV status and the incidence of preterm delivery during COVID-19 period. Albeit previous studies confirmed that HIV-infected women, especially those with uncontrolled infection or without ART, had a higher risk of preterm birth due to immune activation and systemic inflammation [59, 60].

Our findings concur with Mugo and colleagues, who suggests that with effective ART, HIV infection itself may not significantly increase preterm delivery risk [61]. During COVID-19 period, the disruptions in healthcare access and consequent elevations in stress levels may have influenced the risk of preterm birth regardless of the HIV status. Further research is needed to fully understand the complex interplay between HIV, ART, and preterm birth, particularly the specific mechanisms that might lead to an increased risk in some cases.

HIV-infected women also exhibited a non-significant reduction in risk for emergency cesarean section during COVID-19 period, there was a notable trend toward increased elective cesarean deliveries among HIV-infected women. Elective cesareans are the preferred delivery options taken by HIV-infected women to reduce vertical transmission risk [62], particularly when viral suppression is suboptimal [63, 64]. The shift observed during COVID-19 period may reflect a change in hospital policies and a preference for planned interventions to limit both maternal and staff exposure to viral exposure.

Our findings also highlight an association between HIV infection and lower birth weight before the pandemic and higher birth weight during COVID-19 period, albeit non-significant. The analysis showed that HIV-infected women were more likely to deliver infants with low 5 and 10 min APGAR scores during both periods, there was a significant increase in the odds of normal APGAR scores among infants exposed to HIV infants born to HIV-infected mothers during COVID-19 period, compared to HIV-uninfected women.

## Conclusion

This study highlights how the COVID-19 pandemic caused major disruptions to HIV services and prenatal care for pregnant women, particularly those with HIV, which resulted in fewer hospital visits and difficulties in sustaining the best possible care. Modifying management plans such as updated ART regimes helped maintain viral suppression among women with HIV despite these interruptions. Pregnancy outcomes were still influenced by maternal HIV status; before the pandemic, HIV-positive women had different labor patterns and obstetric risks. However, during COVID-19 period, some disparities, like the risk of preterm birth, decreased, perhaps because of multifactorial factors like improved HIV management and changes in healthcare access. Notably, constant maternal mortality and some improved newborn indicators suggested that overall maternal care quality was durable during COVID-19 period, even though poor birth outcomes were still higher among women with HIV. These results demonstrate how crucial it is to continue providing specialized HIV and maternity care during medical emergencies to protect vulnerable groups.

## Data Availability

The data that support the findings of this study are available on request from the corresponding author. The data are not publicly available because of privacy or ethical restrictions.
